# The central melanocortin system and human obesity

**DOI:** 10.1093/jmcb/mjaa048

**Published:** 2020-09-25

**Authors:** Yongjie Yang, Yong Xu

**Affiliations:** 1 USDA/ARS Children’s Nutrition Research Center, Department of Pediatrics, Baylor College of Medicine, Houston, TX, USA; 2 Department of Molecular and Cellular Biology, Baylor College of Medicine, Houston, TX, USA

**Keywords:** obesity, neurons, melanocortin

## Abstract

The prevalence of obesity and the associated comorbidities highlight the importance of understanding the regulation of energy homeostasis. The central melanocortin system plays a critical role in controlling body weight balance. Melanocortin neurons sense and integrate the neuronal and hormonal signals, and then send regulatory projections, releasing anorexigenic or orexigenic melanocortin neuropeptides, to downstream neurons to regulate the food intake and energy expenditure. This review summarizes the latest progress in our understanding of the role of the melanocortin pathway in energy homeostasis. We also review the advances in the identification of human genetic variants that cause obesity via mechanisms that affect the central melanocortin system, which have provided rational targets for treatment of genetically susceptible patients.

## Introduction

Obesity is a serious global health problem due to its increasing prevalence and comorbidities. The World Health Organization (WHO) reported that >650 million adults worldwide were obese in 2016 and 40 million children under the age of 5 were overweight or obese in 2018. In USA, the prevalence of adult obesity was 42.4% in 2017‒2018 according to the Centers for Disease Control and Prevention (CDC). During the past decades, enormous investigations explored mechanisms underlying the regulation of energy homeostasis; many neuropeptides and molecules that regulate energy balance, including those involved in the central melanocortin system, have been identified and recently reviewed (Morton et al., 2006; Xu et al., 2011; Shen et al., 2017; Baldini and Phelan, 2019; Kuhnen et al., 2019). Here, we will review the components of the melanocortin system that have been shown to regulate body weight in both animals and humans.

The central melanocortin system consists of neurons that release endogenous melanocortin ligands and neurons that express the melanocortin receptors (Mcr) (Dores et al., 2016; Shen et al., 2017; Toda et al., 2017). One group of neurons, namely agouti-related protein (Agrp) neurons, is located in the arcuate nucleus of the hypothalamus (ARH) and release orexigenic neuropeptides Agrp and neuropeptide Y (Npy), as well as a neurotransmitter γ-aminobutyric acid (GABA) (Broberger et al., 1998). Agrp is an endogenous melanocortin inverse agonist, which inhibits Mcr (Pritchard et al., 2002; Cone, 2006; Toda et al., 2017). Also located within the ARH are Pomc neurons, which express pro-opiomelanocortin (Pomc) (Elias et al., 1998). The *Pomc* gene transcript can be post-translationally processed to multiple melanocortin ligands, including α-, β-, or γ-melanocyte-stimulating hormone (α-, β-, or γ-Msh), which are endogenous agonists of Mcr (Pritchard et al., 2002; Cone, 2006; Toda et al., 2017). Five subtypes of Mcr (Mc1r, Mc2r, Mc3r, Mc4r, and Mc5r) have been identified, of which Mc3r and Mc4r are expressed primarily in the brain. Through binding with the endogenous melanocortin ligands, these receptors, especially the Mc4r, play a key role in the regulation of energy homeostasis (Cowley et al., 2001; Gautron et al., 2015; Dores et al., 2016; Andermann and Lowell, 2017; Toda et al., 2017). For example, anorexigenic hormones or neurotransmitters such as leptin, insulin, and serotonin activate Pomc neurons, while Agrp neurons are inhibited by these signals, which results in activation of the Mc4r to reduce food intake and/or increase energy expenditure. On the other hand, at the fasted condition, Agrp neurons are activated by the orexigenic hormones, e.g. ghrelin and asprosin, which promotes feeding (Ollmann et al., 1997; Heisler et al., 2006; Atasoy et al., 2012; Zhan et al., 2013; Romere et al., 2016; Duerrschmid et al., 2017). In this review, we will summarize the physiological role of the central melanocortin system in regulating energy homeostasis and its pathophysiological relevance in the development of human obesity.

## The central melanocortin system regulates energy balance

### Pomc and Agrp neurons

The activation of Pomc neurons inhibits food intake and promotes energy expenditure (Mercer et al., 2013; Zhan et al., 2013; Gautron et al., 2015; Dores et al., 2016; Toda et al., 2017), while injury of Pomc neurons leads to obesity (Greenman et al., 2013; Zhan et al., 2013). Mice deficient in the *Pomc* gene are obese and hyperphagic (Yaswen et al., 1999; Challis et al., 2004; Smart et al., 2006). Pomc neurons project to the paraventricular nucleus of the hypothalamus (PVH), where they release the natural agonist of Mc4r, α-Msh, to activate the Mc4r neurons to suppress food intake and increase energy expenditure by modulating the sympathetic outputs to the periphery (Zhang et al., 1994; Ollmann et al., 1997; Cone, 2006; Gautron et al., 2015; Ghamari-Langroudi et al., 2015). The Pomc-originated projections to the PVH require normal functions of the class 3 Semaphorin ligands (Sema3) and their receptors. Pomc-specific deletion of one Sema3 receptor, namely neuropilin-2 receptor (Nrp2), disrupts the Pomc-originated projections to the PVH, which results in weight gain in mice (van der Klaauw et al., 2019).

In contrast to Pomc neurons, Agrp neurons are essential to promote feeding. Transgenic mice with overexpression of Agrp develop obesity (Graham et al., 1997; Ollmann et al., 1997), and intracerebroventricular (ICV) administration of Agrp increases food intake and body weight gain (Fekete et al., 2002). However, mice with germline deletion of the *Agrp* gene do not exhibit hypophagic and lean phenotype (Qian et al., 2002), while ablation of Agrp neurons in adult mice causes loss of appetite and lean phenotype that can result in death due to starvation (Bewick et al., 2005; Gropp et al., 2005; Luquet et al., 2005), suggesting the existence of compensatory mechanisms to regulate energy homeostasis during early development. Food deprivation induces increased expression of Npy and Agrp mRNAs in Agrp neurons (Swart et al., 2002), increases firing activity of Agrp neurons (Takahashi and Cone, 2005), and inhibits Mc4r neurons in the PVH (Cowley et al., 1999; Cowley et al., 2001; Atasoy et al., 2012), which facilitates the conservation of energy storage and also promotes animals to eat when food becomes available again.

### Signals regulating Pomc and Agrp neurons

Pomc and Agrp neurons in the ARH, located alongside the third ventricle and adjacent to the median eminence, are regulated by a broad range of peripheral hormones and neural signals, including leptin, insulin, asprosin, and GABA (Shen et al., 2017).

#### Leptin and insulin

Leptin is a 16-kDa satiety hormone secreted by the white adipose tissue. Most of its physiologic effects are mediated in the brain, including the central melanocortin pathway (Friedman, 2016). Pomc and Agrp neurons both express leptin receptor (LepR) and are the first-order leptin-responsive neurons. Leptin can inhibit Agrp neurons and suppress *Agrp* gene expression. Meanwhile, leptin depolarizes and activates a portion of Pomc neurons, enhancing the *Pomc* gene expression (Schwartz et al., 1997; Mizuno and Mobbs, 1999; Cowley et al., 2001; Friedman, 2016; Shen et al., 2017).

Multiple signal transduction pathways are initiated after the binding of leptin with the long form of LepR. Among these, the Janus kinase 2 (Jak2)‒signal transducer and activator of transcription 3 (Stat3) pathway represents the most critical one to feeding control (Bates et al., 2003; Baldini and Phelan, 2019). Upon binding to leptin, LepR is phosphorylated at Tyr^1138^ by Jak2, which further phosphorylates and activates Stat3. Phosphorylated Stat3 (pStat3) translocates to the nucleus and regulates the transcription of target genes (Baldini and Phelan, 2019). The role of the Tyr^1138^ phosphorylation on the LepR and the contribution of Stat3 signaling to leptin action have been directly addressed by studying a mouse model that harbors the point mutation with the substitution of Tyr^1138^ to Ser. Like *db/db* mice deficient in the *Lepr* gene, *Lepr (S1138)* homozygotes are hyperphagic and obese, which suggests that Stat3 signaling mediates the majority of leptin actions to reduce body weight (Bates et al., 2003).

Leptin induces *Pomc* gene expression and inhibits *Agrp* gene expression, and these effects are at least partly mediated by the activation of Stat3 signaling (Kitamura et al., 2006). pStat3 also initiates a negative feedback pathway by inducing the expression of suppressor of cytokine signaling-3 (*Socs3*), which functions to terminate leptin signaling (Bjorbak et al., 2000). We recently discovered that a transcription co-activator, namely steroid receptor co-activator-1 (SRC-1, encoded by the gene *Ncoa1*), interacts with pStat3 to enhance its transcriptional activity on the *Pomc* gene expression, without affecting the expression of *Socs3*. Thus, SRC-1 enhances the anti-obesity effects of leptin (Yang et al., 2019).

Leptin signaling also crosstalks with insulin signaling pathways to activate phosphatidylinositol 3-kinase (Pi3k) at the level of Jak2 and insulin receptor substrate (Irs) (Kellerer et al., 1997; Carvalheira et al., 2003) and induces the activation of a cation channel, transient receptor potential cation 5 (TrpC5), which is essential for leptin-induced acute activation of Pomc neurons (Hill et al., 2008; Qiu et al., 2014; Gao et al., 2017). The Pi3k pathway promotes the phosphorylation and translocation of forkhead box protein O1 (FoxO1), which promotes *Pomc* gene transcription and increases expression of carboxypeptidase E, an enzyme that regulates the processing of Pomc to α-Msh (Kim et al., 2006; Plum et al., 2009). However, the mapping of insulin- and leptin-responsive Pomc neurons indicates that leptin-activated Pomc neurons are segregated from Pomc neurons that express insulin receptor, which suggests that the crosstalk between leptin and insulin takes place within the Pomc population rather than individual Pomc neurons (Williams et al., 2010). Moreover, the effects of insulin on Pomc neurons still need to be clarified, as studies from independent groups reveal that insulin inhibits Pomc neuronal excitability through the Pi3k pathway and the activation of K_ATP_ channel (Plum et al., 2006; Hill et al., 2008; Williams et al., 2010). In contrast, it has been recently reported that purified insulin depolarizes Pomc neurons via activation of Trpc5 channels, and consistently, ICV-delivered insulin robustly inhibits food intake and activates c-fos expression in Pomc neurons. The authors also identified that Zn^2+^, which is found in insulin formulations at nanomolar concentrations, inhibits Pomc neurons via activation of K_ATP_ channels (Qiu et al., 2014). Unlike the controversial effects of insulin on Pomc neurons, insulin can hyperpolarize Agrp neurons via the K_ATP_ channel (Konner et al., 2007; Varela and Horvath, 2012; Huang et al., 2018). Moreover, in Agrp neurons, phosphorylation and exclusion of FoxO1 from the nucleus reduces the expression of Agrp and Gpr17, a G protein-coupled receptor. ICV injection of Gpr17 agonists induces food intake, which can be blocked by selective deletion of FoxO1 in Agrp neurons (Kitamura et al., 2006; Ren et al., 2012). Sh2b1 is a cytoplasmic adaptor protein involved in leptin and insulin signaling. Sh2b1 binds to numerous protein tyrosine kinases, such as Jak2 in leptin signaling, thereby enhancing the leptin sensitivity; Sh2b1 also binds to receptor tyrosine kinases, e.g. insulin receptor, thereby enhancing the activation of the insulin receptor (Ahmed and Pillay, 2003; Duan et al., 2004; Ren et al., 2005, 2007). Systemic deletion of Sh2b1 results in morbid obesity and severe insulin resistance (IR) (Ren et al., 2005, 2007). Neuron-specific restoration of Sh2b1 not only corrects the metabolic disorders, but also improves leptin and insulin sensitivity (Ren et al., 2007), demonstrating that Sh2b1 is an essential player in the regulation of both leptin and insulin actions in the brain.

#### Asprosin

Asprosin was first discovered as a fasting-induced glucogenic protein hormone that modulates hepatic glucose release (Romere et al., 2016). The fibrillin 1 (*FBN1*) gene encodes a 2871-amino acid-long proprotein (pro-fibrillin), which is cleaved at the C-terminus by the protease furin to generate the mature fibrillin-1, and a 140-amino acid-long, C-terminal cleavage product, named asprosin. Asprosin is believed to be secreted by the white adipose tissue (Romere et al., 2016) and cross the blood–brain barrier to directly activate Agrp neurons and indirectly inhibit Pomc neurons, thereby stimulating food intake (Duerrschmid et al., 2017).

#### Neural signals

Pomc and Agrp neurons in the ARH are also regulated by various neural inputs. For example, Pomc neurons receive inhibitory GABAergic inputs from Agrp neurons (Cowley et al., 2001; Tong et al., 2008). Deletion of the vesicular GABA transporter (Vgat) in Agrp neurons decreases inhibitory tone to Pomc neurons (Tong et al., 2008). Optogenetic studies demonstrate that photostimulation of Agrp neurons results in evoked inhibitory post-synaptic currents in Pomc neurons (Atasoy et al., 2012), although these local inhibitory inputs on Pomc neurons alone are not required for acute feeding effects of Agrp neuron activation (Tong et al., 2008; Atasoy et al., 2012).

Pomc neurons also receive inputs from cholinergic neurons in the dorsomedial hypothalamus (DMH); activation of this DMH to Pomc circuit enhances GABA-mediated inhibitory neurotransmission onto Pomc neurons and promotes feeding (Jeong et al., 2017). On the other hand, Pomc neurons receive strong excitatory inputs from the ventromedial hypothalamus (VMH), which is inhibited by fasting (Sternson et al., 2005). Interestingly, high-fat diet (HFD) feeding also inhibits VMH neuron firing frequency through the insulin-dependent Pi3k activation, which then decreases the activity of Pomc neurons (Klockener et al., 2011). It has been recently reported that Pomc neurons also provide direct inputs to Agrp neurons (Stincic et al., 2018), even though an early examination using channelrhodopsin (ChR2)-assisted circuit mapping failed to identify such projections (Atasoy et al., 2012). During food deprivation, increased level of ghrelin induces the excitatory tone onto Agrp neurons, which can be reversed by leptin through stimulating β-endorphin release from Pomc neurons (Yang et al., 2011). This work, based on the *ex vivo* brain slice recordings, postulates that Pomc neurons can inhibit presynaptic glutamatergic inputs to Agrp neurons. In line with this, recent optogenetic data also show that high-frequency optogenetic stimulation of Pomc neurons enhances the release of β-endorphin, which inhibits postsynaptic Agrp neurons (Stincic et al., 2018); chronic infusion with high dose of β-endorphin suppresses *Agrp* mRNA levels and decreases food intake (Dutia et al., 2012). These data revealed the metabolic role of β-endorphin from Pomc neurons through suppressing Agrp neurons, which may partially explain the previous studies that selective ablation of only the β-endorphin-encoding portion of the *Pomc* gene yields mice that are hyperphagic and overweight (Appleyard et al., 2003).

Agrp neurons receive excitatory glutamatergic inputs, which drives feeding. Deletion of glutamate N-methyl-D-aspartate receptor from Agrp neurons shows markedly reduced body weight, body fat, and food intake (Liu et al., 2012). One such glutamatergic input originates from a subset of glutamatergic neurons from the PVH, and stimulation of these afferent neurons in the PVH markedly activates Agrp neurons and induces intense feeding (Krashes et al., 2014). In addition to inhibiting Agrp neurons directly, leptin can also activate the GABAergic neurons in the DMH, which monosynaptically innervate Agrp neurons, and engage presynaptic potentiation of GABA release to inhibit Agrp neurons (Xu et al., 2018).

### Mc4r neurons in the PVH

Both genetic and pharmacological studies have demonstrated the key role of the Mc4r in regulating food intake and energy balance in rodents. *Mc4r* knockout mice are hyperphagic and obese (Huszar et al., 1997). ICV injection of leptin into obese mice reduces food intake, which can be significantly inhibited in *Mc4r* knockout mice (Marsh et al., 1999). ICV administration of the agonist of the Mc3/4r inhibits feeding and decreases body weight, while the antagonist blocks this inhibition (Fan et al., 1997).

Despite that the Mc4r is broadly expressed in many areas of the brain, Mc4r-expressing neurons in the PVH are thought to be the principle site to promote satiety and mediate leptin's effects on food intake and body weight (Seeley et al., 1997). Notably, the normal development and functions of PVH neurons, including those expressing the Mc4r, require a basic helix‒loop‒helix‒PAS transcription factor, namely single-minded 1 (Sim1) (Kublaoui et al., 2008; Ramachandrappa et al., 2013). Mice lacking *Sim1* die shortly after birth due to the developmental failure of a subset of secretory neurons (such as oxytocin neurons) in the PVH and the supraoptic nucleus (Michaud et al., 1998). The heterozygous *Sim1* knockout mice are viable, but exhibit hypocellular PVH (average <24% cells) and develop early-onset obesity (Michaud et al., 2001). Postnatal *Sim1* deficiency, not affecting the development of PVH neurons, also causes hyperphagic obesity in mice (Tolson et al., 2010). In these mouse models, the reduced expression of oxytocin neuropeptide and Mc4r in the PVH may mediate the hyperphagic obesity (Kublaoui et al., 2008; Tolson et al., 2010). Collectively, these results demonstrate that the effects of Sim1 on energy balance are at least partly attributed to its actions on development and functions of PVH Mc4r neurons.

Re-expression of *Mc4r* in Sim1 neurons (mostly within the PVH) in *Mc4r* knockout mice prevents 60% of the obesity and normalizes the hyperphagic phenotype of the *Mc4r*-null mice, while reduced energy expenditure is unaffected (Balthasar et al., 2005). Re-expression of Mc4r in cholinergic neurons is sufficient to normalize energy expenditure and modestly reduces body weight gain without alteration in food intake (Rossi et al., 2011). The Mc4r neurons in the PVH receive converging innervations containing α-Msh from Pomc neurons and Agrp from Agrp neurons (Cowley et al., 1999). In response to the natural agonist α-Msh, the Mc4r couples to Gαs and induces activation of adenylate cyclase, production of cAMP, and phosphorylation of the transcription factor, cAMP response element-binding protein (Creb) (Gantz et al., 1993; Sarkar et al., 2002). This pathway has been demonstrated to regulate feeding behavior, thermogenesis, and peripheral glucose metabolism (Podyma et al., 2018). Consistent with this, mutations of adult type 3 adenylyl cyclase (*Adcy3*), a member of the adenylyl cyclase family that mediates Gαs signaling, leads to obesity in mice (Wang et al., 2009). Moreover, mice lacking *Creb1* in Sim1 neurons develop obesity (Chiappini et al., 2011). These observations suggest that Mc4r-associated Gαs signaling is essential for energy homeostasis. On the other hand, Agrp inhibits the effect of α-Msh to promote feeding. In addition to the agonist-dependent coupling to Gαs, there is constitutive coupling of Mc4r to Gαs in the absence of agonist, and Agrp acts as inverse agonist to inhibit the constitutive activity of the Mc4r (Nijenhuis et al., 2001). In addition, α-Msh and Agrp can trigger the closure and opening of the inwardly rectifying potassium channel, Kir7.1, to regulate firing activity of Mc4r neurons, and these regulations are independent of the Gαs signaling (Ghamari-Langroudi et al., 2015).

Normal Mc4r functions also require the accessory proteins, belonging to melanocortin receptor accessory protein (Mrap). In particular, melanocortin 2 receptor accessory protein 2 (Mrap2) has been shown to directly interact with the Mc4r and enhance its signaling (Asai et al., 2013). Mrap2 is predominantly expressed in the PVH, especially in Mc4r neurons (Asai et al., 2013; Novoselova et al., 2016; Schonnop et al., 2016; Liang et al., 2018). Mice lacking *Mrap2* develop severe obesity at a young age and heterozygous mice have an intermediate phenotype (Asai et al., 2013). More importantly, mice with selective loss of *Mrap2* only in Sim1 neurons are phenotypically similar to the global knockout mice, suggesting that the role of Mrap2 in the regulation of energy balance is mainly mediated through PVH Mc4r neurons. Further transcriptomic analysis shows significantly decreased expression of *Sim1* in the PVH of *Mrap2*-deficient mice (Novoselova et al., 2016). These findings indicate that Mrap2 is required for normal Mc4r functions and thus contributes to the regulation of energy homeostasis.

Most recently, anaplastic lymphoma kinase (ALK) was identified as a gene associated with the thinness phenotype in humans (Orthofer et al., 2020). Alk is highly expressed in the hypothalamus, especially in the PVH. Alk in the PVH acts as a negative regulator of white adipose tissue lipolysis and sympathetic tone to fine-tune energy homeostasis. Mice with *Alk* deletion specifically in the PVH are resistance to HFD-induced obesity (Orthofer et al., 2020). Given the abundant expression of Mc4r in the PVH, Alk may interact with the Mc4r signaling in PVH neurons to affect body weight balance, a possibility that remains to be examined.

### Other targets of Agrp and Pomc neurons

In addition to the PVH, Agrp neurons also project to the anterior subdivisions of the bed nucleus of the stria terminalis, lateral hypothalamus area (LHA), and the parabrachial nucleus (PBN) (Wu et al., 2009; Betley et al., 2013; Steculorum et al., 2016). Optogenetic activation of these Agrp-originated circuits evokes increased feeding behavior that is comparable to the somatic activation of Agrp neurons.

Pomc neurons also project to the LHA to regulate food intake and body weight (Elias et al., 1999). Additionally, Pomc neurons project to the VMH and control food intake through the Mc4r-mediated regulation of brain-derived neurotrophic factor (*Bdnf*) expression in the VMH (Xu et al., 2003). Bdnf is a secreted neurotrophin highly expressed in the VMH (Xu et al., 2003) and PVH (An et al., 2015). Bdnf, as well as its receptor tropomyosin-related kinase B (TrkB, encoded by the *Ntrk2* gene), plays an essential role in regulating appetite and energy balance, as chronic ICV delivery of Bdnf inhibits body weight gain (Liao et al., 2012; Waterhouse and Xu, 2013). Furthermore, mice with decreased *Bdnf* expression show hyperphagia and obesity, which can be reversed by central infusion of Bdnf (Kernie et al., 2000; Unger et al., 2007). In addition, actions of leptin to activate hypothalamic neurons and inhibit food intake are compromised in *Bdnf* mutant mice (Liao et al., 2012). Ablation of Bdnf-expressing neurons in the PVH largely blunts the effects of leptin to promote sympathetic innervation to adipose tissue in mice (Wang et al., 2020). Consistently, Bdnf infusion into the brain can suppress the hyperphagia and body weight gain in *Mc4r* knockout mice, which suggests that Bdnf at least partly mediates Mc4r actions to regulate energy balance (Xu et al., 2003). Moreover, mice with *Bdnf* ablation in the PVH develop hyperphagia, impaired thermogenesis, and severe obesity (An et al., 2015). Similarly, mice with reduced TrkB expression (25% of the normal level), due to an *Ntrk2* gene mutation, exhibit hyperphagic and obese phenotypes when fed with HFD (Xu et al., 2003), and central infusion of TrkB agonists reduces food intake and body weight in these mice (Tsao et al., 2008). *Ntrk2* deletion in the DMH leads to modest hyperphagia and obesity (Liao et al., 2019), and the deletion of *Ntrk2* gene in the PVH leads to severe hyperphagic obesity (An et al., 2020). In addition, the neurocircuit from the TrkB-expressing neurons in the PVH to the VMH and the lateral PBN are reported to suppress appetite (An et al., 2020).

## Genetic variants affecting the central melanocortin system cause obesity in humans

Human genetic studies (including GWAS) have identified common and rare genetic or epigenetic variants that are associated with human obesity (Farooqi and O'Rahilly, 2000; Locke et al., 2015; Wahl et al., 2017). Strikingly, most of the obesity-associated human variants affect genes that are abundantly expressed in the central nervous system (Locke et al., 2015). This unique pattern strongly suggests that dysfunctions of the brain play essential roles in the development of human obesity. With the critical role of the central melanocortin system in regulating energy balance, it is not surprising that even a monogenic mutation in the melanocortin pathway often results in severe, early-onset obesity in humans. Here, we will review advances in the identification of human genetic variants that cause body weight imbalance via mechanisms that affect the central melanocortin system.

### POMC

*POMC* gene mutation in humans was first reported in 1998; then, more cases of *POMC* deficiency were identified (Krude et al., 1998; Farooqi et al., 2006). All the patients, with the deficiency of *POMC* gene-derived peptides, presented severe, early-onset obesity associated with hyperphagia, although the number of identified human cases is extremely low (Farooqi and O'Rahilly, 2008). Comparing to the loss of POMC-derived peptides, even the loss of one copy of the *POMC* gene predisposes to obesity in humans (Farooqi and O'Rahilly, 2008). Moreover, a variety of heterozygous point mutations in the *POMC* gene resulting in loss of function of α-MSH or β-MSH are reported to increase the risk of obesity. For example, children carrying the Tyr221Cys variant in the region encoding β-MSH, which impairs its ability to activate the MC4R, are hyperphagic and obese (Lee et al., 2006; Farooqi and O'Rahilly, 2008).

In addition to the *POMC* gene itself, other genetic variants may also cause human obesity through negatively affecting the expression of POMC. For example, we identified a group of *SRC-1* (encoded by the *NCOA1* gene) variants from early-onset, severely obese children (Yang et al., 2019). In cultured cells, we showed that these mutated SRC-1 proteins compete and disrupt the normal function of wild-type SRC-1 protein and impair leptin-induced *POMC* expression. Importantly, a knock-in mouse model mimicking one of these human variants (*SRC-1^L1376P/+^*) develops hyperphagia and obesity (Yang et al., 2019). In addition, leptin-induced depolarization of Pomc neurons and *Pomc* gene expression are significantly reduced in these mice (Yang et al., 2019). These data support the notion that the loss-of-function *SRC-1* variants result in obesity in humans likely due to impaired functions of the central melanocortin system. Furthermore, the Semaphorin 3 signaling has been reported to promote the development of Pomc projections to the PVH, and loss of a Sema3 receptor, namely Nrp2, causes obesity in mice (van der Klaauw et al., 2019). Interestingly, multiple missense mutations have been identified in genes encoding SEMA3 ligands and their receptors in patients with severe, early-onset obesity, which likely underlie the development of weight gain (van der Klaauw et al., 2019).

### AGRP

Mutation screening in human genes has revealed some single-nucleotide polymorphisms (SNPs) in the *AGRP* gene that show potential linkage to body weight dysregulations (Ilnytska and Argyropoulos, 2008). The T allele of the SNP −38C>T (rs5030981) has been associated with lower promoter activity, low body fatness, and resistance to developing type 2 diabetes (Mayfield et al., 2001; Argyropoulos et al., 2003; Bai et al., 2004; Bonilla et al., 2006). The SNP +79G>A (rs34018897) is implicated to be associated with reduced resting metabolic rate and increased fat mass (Sözen et al., 2007). The SNP 131-42C>T (rs11575892), located in the second intron of the human *AGRP* gene, is found from the screening of 95 patients with severe obesity, and heterozygotes at this position possess significantly higher body mass index (BMI) in the Latvian population (Kalnina et al., 2009).

One most investigated SNP 199G>A (rs5030980) is located in the coding region of *AGRP* and leads to amino acid substitution, Ala67Thr (Argyropoulos et al., 2002). Individuals homozygous for Ala67Ala have higher BMI and increased body fat (Argyropoulos et al., 2002; Li et al., 2014), whereas those homozygous for Thr67Thr have lower BMI and body fat (Marks et al., 2004). Interestingly, in Dutch, Ala67Ala is associated with increased BMI only in men but not in women (van Rossum et al., 2006), suggesting a possible sexual dimorphism in the functions of this SNP.

While no report links the SNPs located within the active form of AGRP (amino acids 83–132) and the metabolism in humans, some SNPs in this region have been deposited in the NIH Variation Viewer database (Ericson and Haskell-Luevano, 2018). Most recently, these SNPs have been tested *in vitro* for potential impacts on cellular signaling and functions of the MC4R. All the SNPs tested result in at least a 10-fold decreased potency in inhibiting the MC4R, suggesting that SNPs may impact AGRP functions (Koerperich et al., 2020).

### Leptin and leptin receptor

Leptin-deficient mice display hyperphagic and obese phenotype, and the obese gene (*Lep*) mutation in mice was identified in 1950. However, the reason for a single-gene mutation of the *Lep* gene resulting in profound obesity and diabetes was unknown until the cloning of leptin in 1994 (Zhang et al., 1994). Since then, multiple forms of *Lep* gene mutations have been identified in patients. These mutations include homozygous frameshift, as well as nonsense and missense mutations, which result in an inability to produce the leptin protein. Humans with *LEP* deficiency are obese and diabetic, although such mutations are rare in the population (Montague et al., 1997; Farooqi and O'Rahilly, 2008). The administration of leptin to leptin-deficient mice can rescue hyperphagia and obesity. Similarly, daily subcutaneous treatment of leptin to patients with *LEP* gene deficiency also corrects obesity, which is largely attributable to changes in energy intake (Halaas et al., 1995; Pelleymounter et al., 1995; Farooqi et al., 1999). The therapeutic response to leptin in humans with *LEP* deficiency confirms the importance of leptin in the regulation of human body weight.

Similar to *Lep* gene, a single *Lepr* gene mutation (leptin receptor-deficient mice, *db/db*) also leads to severe obesity (Hummel et al., 1966). In 1996, several groups reported that *Lepr* has multiple splicing forms and the long form of the leptin receptor harbors mutation in the intracellular domain that affects the intracellular signaling in *db/db* mice (Chen et al., 1996; Chua et al., 1996; Lee et al., 1996). In humans, homozygous mutation of the *LEPR* gene results in a truncated leptin receptor lacking both transmembrane and intracellular domains and the patients show early-onset morbid obesity (Clement et al., 1998). Overall, up to 3% of patients with severe obesity have been found to harbor mutations in the *LEPR* gene that are associated with a loss of function in the protein (Farooqi and O'Rahilly 2008).

In addition to the deletion of *LEP* and *LEPR*, the polymorphisms of these two genes are also extensively studied (Marti et al., 2009; Labayen et al., 2011; Paolini et al., 2016; Ren et al., 2019). However, the associations between these polymorphisms and human obesity are still controversial. For example, for the commonly studied *LEPR* SNP rs8179183, it has been reported that there is a significant association with obesity in Chinese Han and European adolescents (Labayen et al., 2011; Ren et al., 2019), but no significant association in Spanish adults (Marti et al., 2009).

Recently identified 16p11.2 deletions encompass several genes including *SH2B1*, which is known to be involved in leptin and insulin signaling (Bochukova et al., 2010). Systemic deletion of *Sh2b1* in mice results in morbid obesity and severe IR (Ren et al., 2005, 2007). Similarly, patients with deletion of 16p11.2 are associated with highly penetrant familial severe early-onset obesity (Bochukova et al., 2010). Although the contribution of other genes or non-coding genetic material cannot be excluded, the phenotype is consistent with the role of SH2B1 in human energy homeostasis.

### Asprosin

The neonatal progeroid syndrome (NPS) was first described in 1977 (Rautenstrauch and Snigula, 1977; Romere et al., 2016). The NPS patients are extremely lean and have significantly less food intake (Romere et al., 2016; Duerrschmid et al., 2017). Whole-exome sequencing identified mutations from seven NPS patients, which are clustered around the cleavage site of the pro-fibrillin protein, leading to the truncated mutations and heterozygous ablation of the C-terminal cleavage product, asprosin, in patients (Romere et al., 2016).

Asprosin can activate Agrp neurons and promote feeding, while neutralizing asprosin with an antibody reduces food intake in mice (Duerrschmid et al., 2017). Recent studies have discovered the crucial role of asprosin in association with human obesity. It has been reported that circulating asprosin levels are significantly higher in obese adults and children than in non-obese subjects, and children with IR have higher asprosin levels than non-IR group (Wang et al., 2019a, b). Furthermore, asprosin level is associated with obesity, as the amount increases in accordance with the increasing BMI; on the other hand, there is also a relationship between the underweight and asprosin, because the amount decreases with the decreasing BMI (Ugur and Aydin, 2019). However, contradictory results were also reported that serum asprosin concentrations are significantly lower in obese children compared to normal-weight children and the level is negatively associated with BMI (Long et al., 2019). Further investigations are required for the clarification of conflicting roles of asprosin in the human obesity.

### MC4R and associated molecules

*Mc4r* knockout mice display hyperphagia and severe obesity, while the loss of one *Mc4r* allele results in an intermediate obese phenotype, suggesting a gene dosage effect of *Mc4r* expression on body weight regulation (Huszar et al., 1997; Balthasar et al., 2005). Similar to mouse mutations, the heterozygous mutations in human *MC4R* gene are associated with severe, early-onset obesity (Vaisse et al., 1998; Yeo et al., 1998). Currently, *MC4R* mutations represent the most common monogenic cause of severe obesity in humans, accounting for ∼5% of obese patients, particularly those with early-onset obesity (Farooqi et al., 2003; Larsen et al., 2005; Farooqi and O'Rahilly, 2008). Furthermore, there are 376 single-nucleotide variants (SNVs) and 189 copy number variants reported in the *MC4R* gene region (Fairbrother et al., 2018). These mutations may disrupt ligand binding, affect the cell surface expressing, Gαs signaling cascade, and cAMP activation, and lead to the biased downstream signal transduction (Farooqi and O'Rahilly, 2008; Kuhnen et al., 2019). One recently identified nonsense p.Tyr35Ter *MC4R* SNV (rs13447324) is present in ∼1 in 5000 individuals and leads to ∼7 kg higher body weight for a 1.7-m-tall person (Turcot et al., 2018). Moreover, most recent genetic studies in >0.5 million people have identified that the β-arrestin-biased *MC4R* variants are associated with significantly lower BMI, lower risk of obesity, and its cardio-metabolic complications in general population (Lotta et al., 2019).

Since most patients are heterozygous *MC4R* mutation carriers, it is possible that MC4R agonists can be used to reduce body weight in these individuals. During the past decades, a variety of peptides and small chemical MC4R agonists have been developed and shown to reduce food intake and body weight in rodents (Goncalves et al., 2018). However, these beneficiary effects are frequently associated with cardiovascular side effects due to the MC4R-related sympathetic activation (Fani et al., 2014; Goncalves et al., 2018; Kuhnen et al., 2019). Setmelanotide, a new generation of synthetic 8-amino acid cyclic MC4R agonist peptide, can effectively induce biased signaling of the MC4R, thereby reducing appetite and leading to weight loss without adverse effects in heart rate or blood pressure (Chen et al., 2015; Collet et al., 2017). It has been shown to suppress food intake and body weight in obese mice and monkeys (Collet et al., 2017), and clinical treatment on three severely obese *LEPR*-deficient individuals shows substantial and durable reductions in hyperphagia and body weight over 45‒61 weeks (Clement et al., 2018). Currently, setmelanotide is in the phase 3 clinical trial for various human obesity syndromes, including *POMC* deficiency, *LEPR* deficiency, Bardet–Biedl syndrome, Alström syndrome, and others with impaired MC4R pathway (Kuhnen et al., 2019; Sharma et al., 2019).

In mice, Sim1 is required to mediate normal development and functions of PVH Mc4r neurons, and therefore loss of *Sim1* causes obesity in animals at least partly due to impaired melanocortin signaling (Michaud et al., 1998, 2001; Tolson et al., 2010). Similar to mouse models, patients with chromosomal deletions involving 6q16.2 resulting in *SIM1* gene deletion develop early-onset obesity (Villa et al., 1995; Faivre et al., 2002; Wang et al., 2008). A patient with severe, early-onset obesity is associated with the balanced 1p22.1 and 6q16.2 chromosome translocation, which disrupts one allele of the *SIM1* gene (Holder et al., 2000). A study in the Pima Indian population also indicates that common variation in *SIM1* is associated with human BMI (Traurig et al., 2009). Moreover, the sequencing of *SIM1* coding region in 2100 patients with severe, early-onset obesity and 1680 controls has identified 13 heterozygous variants. Variant carriers exhibited increased *ad libitum* food intake (Ramachandrappa et al., 2013). These clinical findings support the effects of SIM1 on the regulation of energy homeostasis in humans.

Mrap2 is required for normal Mc4r functions (Asai et al., 2013) that contribute to the regulation of energy homeostasis (Novoselova et al., 2016). Consistently, human *MRAP2* variants were identified in obese individuals recruited to the Genetics of Obesity Study and in the Swedish obese children’s cohort. Four rare heterozygous variants (N88Y, L115V, R125C, and E24X) were identified and one of the variants (E24X) is clearly disruptive (Asai et al., 2013). Further study shows that N88Y and R125C have impaired capability to enhance α-MSH-induced MC4R activation (Liang et al., 2018). Similarly, two more novel MRAP2 variants (A137T and Q174R) were detected in an individual with extreme obesity, and the Q174R mutant loses its potentiating effect on MC4R (Schonnop et al., 2016). Most recently, a large-scale sequencing study of *MRAP2* in 9418 people revealed 23 rare heterozygous variants associated with increased obesity risk in both adults and children (Baron et al., 2019). Functional assessment of each variant shows that loss-of-function *MRAP2* variants are pathogenic for monogenic hyperphagic obesity (Baron et al., 2019). Taken together, these findings suggest that the decreased MC4R activity caused by the loss-of-function mutations in the *MRAP2* gene contributes to obesity in human carriers.

A GWAS study on metabolically healthy thin individuals in an Estonian cohort identified the genetic variants in ALK associated with thinness (Orthofer et al., 2020). Two top ALK variants (indels rs568057364 and rs202021741) and four downstream variants (rs12990552, rs10495771, rs55737023, and rs7578465) within the ALK locus are associated with human BMI. Experimental enhancer assays in a human neuroblastoma cell line support the notion that the top ALK variants might be located in a regulatory region and the region around rs568057364 has enhancer activity. Alk in mouse is mainly expressed in the hypothalamus, especially in the PVH, which is also true for humans, and the ALK variants may affect the expression of ALK in specific brain regions. The consequence of the intronic ALK variants associated with human metabolism still requires further investigations (Orthofer et al., 2020).

### BDNF and TRKB

The identification of rare genetic mutations in the *BDNF* and *NTRK2* genes provides further evidence to link BDNF signaling with human obesity. The first reported rare mutation in *NTRK2* was from an 8-year-old boy who harbored a heterozygous missense mutation resulting in a Y722C substitution and showed developmental syndrome, hyperphagia, and severe obesity (Yeo et al., 2004). Similarly, a child with a *de novo* chromosomal inversion that disrupted the expression of one *BDNF* allele developed obesity and neurobehavioral phenotypes (Gray et al., 2006). As the loss of one allele of *BDNF*, this patient also had much lower blood BDNF level, which suggests that BDNF level may be associated with development of childhood obesity. Consistent with this, a subset of patients with WAGR syndrome (Wilms tumor, aniridia, genitourinary abnormalities, and mental retardation) were associated with the *BDNF* gene deletion and altered BDNF expression and also developed childhood obesity (Han et al., 2008). It was also found that decreased plasma BDNF levels are associated with birth weight and BMI in morbidly obese children (Araki et al., 2014). However, this relationship between serum BDNF levels and obesity is still controversial. It has been reported that circulating BDNF levels are decreased in young non-obese subjects with low insulin sensitivity (Karczewska-Kupczewska et al., 2011) and increased BDNF is associated with type 2 diabetes mellitus (Suwa et al., 2006). Multiple GWAS studies have identified seven SNPs in or near the *BDNF* gene (rs4074134, rs4923461, rs925946, rs10501087, rs6265, rs10767664, and rs2030323) that are associated with human obesity (Waterhouse and Xu, 2013). One of the most extensively studied SNPs is rs6265 that leads to a Val66Met mutation of the pro-BDNF, which is significantly correlated with childhood obesity in European (Zhao et al., 2009) and Chinese populations (Wu et al., 2010).

## Conclusions

**Figure 1:**
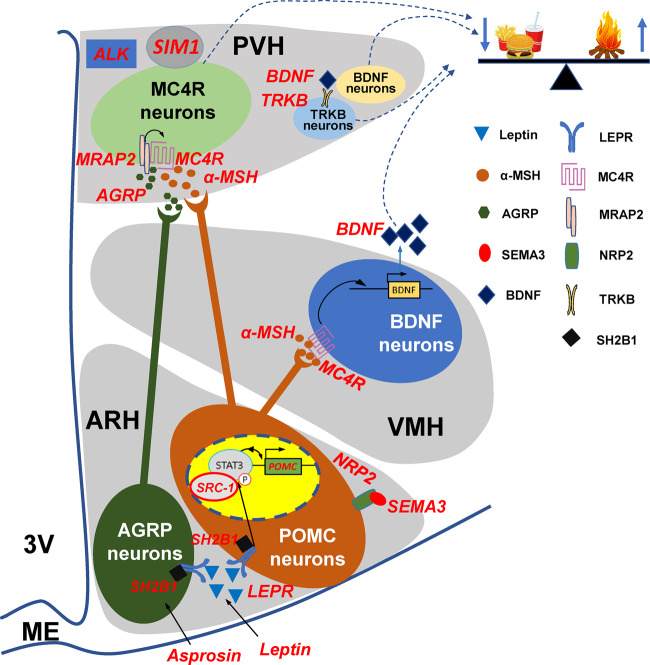
A simplified summary of the central melanocortin pathway including the key components that are affected by known human genetic variants (red in text). 3V, third ventricle; α-MSH, α-melanocyte-stimulating hormone; AGRP, agouti-related peptide; ALK, anaplastic lymphoma kinase; ARH, arcuate nucleus of the hypothalamus; BDNF, brain-derived neurotrophic factor; LEPR, leptin receptor; MC4R, melanocortin 4 receptor; ME, median eminence; MRAP2, melanocortin 2 receptor accessory protein 2; NRP2, neuropilin-2 receptor; POMC, pro-opiomelanocortin; PVH, paraventricular nucleus of the hypothalamus; SEMA3, semaphorin 3; SIM1, single-minded 1; SRC-1, steroid receptor co-activator-1; STAT3, signal transducer and activator of transcription 3; TRKB, tropomyosin-related kinase B; VMH, ventromedial hypothalamus.

Several decades of scientific research on obesity have contributed dramatically to our understanding of the genetic basis and the neuroendocrine pathways that mediate the regulation of body weight homeostasis (Tables 1 and 2). As we discussed above, a variety of genetic variants responsible for human obesity disrupt the development of melanocortin neurons, production of melanocortin ligands, and upstream or downstream signaling of melanocortin neurons, which highlights the critical importance of the central melanocortin system in the regulation of energy balance in humans (Figure 1).

**Table 1:** Mouse genes that regulate the melanocortin system and body weight.

Gene	Genetic manipulation	Body weight phenotype	References
*Pomc*	Deletion	Severe early-onset obesity	Yaswen et al. (1999); Challis et al. (2004); Smart et al. (2006)
*Agrp*	Overexpression	Severe early-onset obesity	Graham et al. (1997); Ollmann et al. (1997)
*Leptin*	Deletion	Severe early-onset obesity	Zhang et al. (1994)
*LepR*	Deletion	Severe early-onset obesity	Chen et al. (1996); Chua et al. (1996); Lee et al. (1996)
*Mc4r*	Deletion	Severe early-onset obesity	Huszar et al. (1997)
*Sim1*	Heterozygous deletion	Severe early-onset obesity	Michaud et al. (2001); Tolson et al. (2010)
*Mrap2*	Deletion	Severe early-onset obesity	Asai et al. (2013)
*Fbn1*	Mutation causing asprosin deficiency	Lean	Duerrschmid et al. (2017)
*Sema3/ Nrp2*	Deletion in Pomc neurons	Mild obesity	van der Klaauw et al. (2019)
*Ncoa1 (SRC-1)*	Loss-of-function mutation; deletion in Pomc neurons	Mild obesity	Yang et al. (2019)
*Sh2b1*	Deletion	Severe obesity	Ren et al. (2005, 2007)
*Bdnf*	Heterozygous deletion	Severe obesity	Kernie et al. (2000); Unger et al. (2007)
	Deletion in PVH	Severe obesity	An et al. (2015)
*Alk*	Deletion in PVH	Lean	Orthofer et al. (2020)
*Ntrk2*	Mutation	Severe early-onset obesity	Xu et al. (2003)
	Deletion in PVH		An et al. (2020)

**Table 2:** Human genes that affect the melanocortin system and are associated with body weight.

Gene	Genetic function	Body weight phenotype	References
*POMC*	Loss-of-function mutations	Severe early-onset obesity	Krude et al. (1998); Farooqi et al. (2006); Lee et al. (2006); Farooqi and O'Rahilly (2008)
*NCOA1 (SRC-1)*	Loss-of-function mutations	Severe early-onset obesity	Yang et al. (2019)
*AGRP*	SNP in non-coding or coding regions	Obesity or leanness	Ilnytska and Argyropoulos (2008)
*LEPTIN*	Loss-of-function mutations	Severe early-onset obesity	Montague et al. (1997); Farooqi and O'Rahilly (2008)
*LEPR*	Loss-of-function mutations	Severe early-onset obesity	Clement et al. (1998)
*FBN1*	Mutation causing asprosin deficiency	Extreme leanness	Romere et al. (2016); Duerrschmid et al. (2017)
*SH2B1*	Deletion	Severe early-onset obesity	Bochukova et al. (2010)
*MC4R*	Loss-of-function mutations	Severe early-onset obesity	Vaisse et al. (1998); Yeo et al. (1998)
	Variants/various effects	Various effects	Farooqi and O'Rahilly (2008); Fairbrother et al. (2018); Kuhnen et al. (2019)
*SIM1*	Deletion	Severe early-onset obesity	Villa et al. (1995); Faivre et al. (2002); Wang et al. (2008)
	Loss-of-function mutations	Severe early-onset obesity	Ramachandrappa et al. (2013)
*MRAP2*	Loss-of-function mutations	Severe obesity	Asai et al. (2013); Baron et al. (2019)
*BDNF*	Deletion	Severe early-onset obesity	Gray et al. (2006)
	SNP/variants	Obesity	Waterhouse and Xu (2013)
*ALK*	Variants/unknown	Leanness	Orthofer et al. (2020)
*NTRK2*	Loss-of-function mutations	Severe early-onset obesity	Yeo et al. (2004)

It is important to emphasize that the combination of human research and basic animal neuroendocrinology studies has significantly advanced the field by identifying novel genetic obesity syndromes in humans and revealing the underlying dysregulated neuroendocrine functions (Montague et al., 1997; Farooqi et al., 2007; van der Klaauw et al., 2019). These studies all take advantage of the combined human and mouse genetics to provide compelling evidence for the cause of a human disease and the underlying mechanisms. Since most of the obesity-associated human variants affect genes that are enriched in the brain (Locke et al., 2015), we suggest that if we can bring together the diverse expertise in human obesity research and basic neuroendocrinology, much more can be learned about obesity development, mechanisms, and treatment. Indeed, several of these genetic disorders are now treatable (Farooqi et al., 1999). Recent clinical trials have shown that patients with genetic syndromes that impair the central melanocortin system can be treated with an Mc4r agonist (Potel et al., 1988; Kuhnen et al., 2016; Collet et al., 2017; Clement et al., 2018). The CRISPR-mediated activation (CRISPRa) gene therapy can be used to upregulate the remaining functional copy of the haploinsufficient gene using the endogenous regulatory elements (Matharu et al., 2019). The CRISPRa targeting of the *Sim1* promoter or its distant hypothalamic enhancer increases its expression from the endogenous functional allele and rescues the obesity phenotype in *Sim1* heterozygous mice. Similarly, injection of CRISPRa-recombinant adeno-associated virus into the hypothalamus of *Mc4r*-haploinsufficient mice leads to reversal of the obesity phenotype. This work provides a framework to further develop the CRISPRa as a potential tool to treat gene dosage-related obesity (Matharu et al., 2019).

## Funding

The authors are supported by the National Institutes of Health (NIH) grants (DK113954, DK117281, DK115761, and USDA/CRIS 3092-51000-064-01S).

**Conflict of interest:** none declared. 
